# Influence Mechanism of Energy Efficiency Label on Consumers’ Purchasing Behavior of Energy-Saving Household Appliances

**DOI:** 10.3389/fpsyg.2021.711854

**Published:** 2021-10-18

**Authors:** Guo Si-dai, Lu Cheng-Peng, Li Hang, Zhu Ning

**Affiliations:** ^1^School of Economics and Management, Southwest University of Science and Technology, Mianyang, China; ^2^Institute of County Economic Development and Rural Revitalization Strategy, Lanzhou University, Lanzhou, China

**Keywords:** energy efficiency label, energy-saving household appliances, purchasing behavior, influence mechanism, intermediary effect

## Abstract

Mandatory energy efficiency label is an effective way to change consumers’ consumption habits and guide them to buy energy-saving appliances. However, few studies concerned about the impact of energy efficiency label on consumers’ purchasing behavior. Based on the theory of planned behavior (TPB), social cognitive theory and signaling theory, this paper constructs a theoretical model of the effect of the energy label on consumers’ purchasing behavior of energy-saving household appliances. The survey data of 396 household appliance consumers in Mianyang City, China, are collected by the interception method, and the theoretical model is tested by structural equation modeling (SEM). Empirical results of this study indicate that consumers’ cognition and perceived value of energy efficiency label significantly affect label trust. Perceived value has a significant impact on consumers’ purchasing behavior of energy-saving appliances, while label cognition and label trust indirectly influence consumers’ purchasing behavior through the intermediary variable of purchase intention. External environmental factors such as publicity and education as well as subjective norms affect consumers’ actual purchasing behavior through the intermediary effect of purchase intention. This study provides important insights into the policy intervention measures to promote consumers’ purchasing behavior of energy-saving appliances.

## Introduction

Energy consumption of household appliances is an important component of household energy consumption in China. About 70% of household carbon dioxide emissions result from household appliances, of which refrigerators, air conditioners and televisions account for 50% (Guo et al., 2018). By guiding consumers to buy energy-saving appliances, household energy consumption can be effectively reduced, which is an effective method to achieve the target of energy conservation and emission reduction (Song et al., 2019a; Wang Z. et al., 2019; Zhang et al., 2020). Therefore, governments all over the world, including China, have realized the importance of promoting energy-saving appliances and guiding consumers to use them, and have issued various supportive policies, among which the most effective is the informative energy efficiency label program. In 1975, the United States first proposed the energy efficiency labeling system in The Energy Policy and Conservation Act. Canada began to implement the mandatory energy efficiency labeling (Energy Guide) in 1978. China’s household appliance industry formally implemented the mandatory energy efficiency labeling system in 2005, and issued a new version of The Energy Efficiency Label Management Measures in 2016. By July 2020, the catalog of products with energy efficiency label has been updated to the 15th batch. The implementation of this system has accelerated the elimination of low efficiency household appliances. The application of energy efficiency label is becoming more and more popular in the home appliance market all over the world (Weil and McMahon, 2003). Most of the research results in this field show that energy efficiency label can have a positive impact on consumers’ home appliance purchasing behavior and play a certain role in reducing energy consumption (Shen and Saijo, 2009; Sammer and Wüstenhagen, 2010; Ward et al., 2011). Therefore, energy efficiency label is a common tool to reduce energy consumption of household appliances in many countries around the world (Mahlia and Saidur, 2010; Anna et al., 2018), which provides additional information about product characteristics for consumers and especially plays a crucial role in shaping consumers’ choice of energy-saving electrical products (Zainudin et al., 2014).

Energy efficiency label provide consumers with environmental and energy information related to home appliance products and services, aiming to help consumers compare and choose more energy-efficient products (Brazil and Caulfield, 2017). At the same time, household appliance enterprises can also increase the credibility of information through the third-party certification of energy efficiency label (Vanclay et al., 2011). Studies have shown that consumers in developed countries such as Germany and Italy prefer to buy energy-efficient appliances with higher energy efficiency grades (Topolansky Barbe et al., 2013; Tan et al., 2016). Sammer and Wüstenhagen’s research has found that consumers prefer to choose energy-saving products with higher energy efficiency when purchasing home appliances (Sammer and Wüstenhagen, 2010). The Energy Star labeling program in the United States has been successful since its implementation. Scholars have studied consumers’ intention to buy refrigerators under the Energy Star policy, and found that consumers are willing to pay 249.8–349.3 US dollars for refrigerators with energy star label (Murray and Mills, 2011). A study based on a large online retailer in Switzerland has found that the energy efficiency label can increase the sales of energy-saving appliances, and that the impact of different designs of label on consumers’ purchase decisions is similar (Marcel and Renate, 2018). Chinese consumers are willing to buy energy-saving household appliances with higher energy efficiency, but they have different choices toward conditioners and washing machines, that is, they tend to buy energy-saving refrigerators rather than energy-saving air-conditioners (Shen and Saijo, 2009).

Whether consumers concerned about energy efficiency label is a key factor affecting their purchasing behavior of energy-saving appliances. When consumers trust the energy efficiency label of products and they have had the intention to purchase energy-saving appliances, they often pay more attention to energy efficiency labels (Issock et al., 2018). Similarly, some research results show that the decisive factor of the effectiveness of the Energy Star labeling program is whether consumers pay attention to energy efficiency labels. If consumers are willing to know about them, they are likely to respond to the information on the labels and change their purchasing behavior (Murray and Mills, 2011). The results of an online survey in Brunei also show that the energy efficiency labeling system for air conditioning systems can encourage manufacturers to improve their system energy efficiency, and the energy efficiency labeling system which is developed based on consumers’ concern has a significant impact on reducing the overall energy consumption of the country (Abas and Mahlia, 2018). When consumers are willing to pay attention to energy efficiency label and can respond to the information on them, they prefer to buy energy-saving appliances with higher energy efficiency grade and level. However, a large-scale survey of more than 20,000 German households by Mills and Schleich (2010) shows that when consumers lack an understanding of energy efficiency label, there may be large deviations in the estimation of the utilization rate of energy-saving appliances and their potential determinants. Consumers’ response to energy efficiency label is also affected by the label type. Generally speaking, consumers pay more attention to and trust in the mandatory labeling scheme, and they are more likely to change their purchasing behavior in response to the information on energy efficiency label. In this case, energy efficiency label can help consumers make the best choice for household appliances with different energy efficiency grades (Bernard et al., 2015).

A very small number of scholars have come up with research that contradicts previous studies. Zainudin’s study found that energy efficiency label is negatively correlated with green purchasing behavior, and energy efficiency label has no effect on encouraging consumers to deliver good information in purchasing decisions (Zainudin et al., 2014). Similar to this research result, research evidence from South Africa shows that most consumers do not give priority to energy efficiency label when purchasing products, but take other factors into consideration (Dreyer et al., 2016).

Most studies have shown that energy efficiency label can guide consumers to buy energy-saving appliances. However, the formation of consumers’ decision-making behavior is a complex process. Issues such as how energy efficiency label change consumers’ purchasing behavior, what are the deep-seated psychological reasons of consumers’ purchasing behavior of energy-saving appliances and what is the internal influence mechanism are worthy of further study. There are a lot of researches on the influencing factors of green consumption behavior and energy-saving behavior in academia. However, there are few achievements of researches based on energy efficiency label’ influence on Chinese consumers’ intention and behavior of energy-saving consumption. Therefore, based on the theory of planned behavior (TPB), social cognitive theory and signaling theory, this paper uses the structural equation model to explore the internal influence mechanism of consumers’ purchasing behavior of energy-saving household appliances.

The rest of the paper is organized as follows. Section “Theoretical Framework, Variables, and Hypothesis” introduces the theoretical framework, variables and research hypothesis used in this paper. Section “Methodology” and “Results” demonstrates the methodology and the empirical results respectively. Section “Discussion” presents the discussion. Finally, the conclusions, policy implications, limitations and perspectives are drawn in Section “Conclusions, Policy Implications, Limitations, and Perspectives.”

## Theoretical Framework, Variables, and Hypothesis

### Theoretical Basis

#### Theory of Planned Behavior

Theory of Planned Behavior is mainly used to predict and explain the occurrence of human behaviors in specific environments (Ajzen, 1991). This theory is now one of the most widely used social psychological models to understand and predict human behavior patterns. It is based on the assumption that human beings act in the way of rational man. The core element of this theory is whether a person intends to perform a given behavior (Conner and Armitage, 1998).

TPB includes five core elements, such as behavior attitude, subjective norm, perceived behavior control, behavior intention and actual behavior. Behavioral attitude refers to a person’s positive or negative attitude toward the implementation of a certain action. The formation of attitude can be explained from two levels: the important beliefs of the individual’s behavior results and the evaluation of the results (Fishbein and Ajzen, 1975). Subjective norm refers to the social pressure of individuals when they take a particular behavior. This kind of pressure mainly comes from important salient individuals or groups, such as parents, spouses, friends, colleagues, etc. Subjective norm is the sum of normative belief and motivation to comply. Perceptual behavior control is the degree to which an individual perceives that it is easy or difficult to perform a particular behavior. It can influence the actual behavior indirectly by controlling behavior intention, and can also be used to predict the occurrence of actual behavior (Xu et al., 2013). Behavioral intention is the willingness of individual’s subjective probability when they take a particular behavior (Fishbein and Ajzen, 1975). Behavior intention is the necessary process of any behavior performance, and it is the decision before the behavior appears. Actual behavior is the actual behavior taken by individuals.

TPB believes that an individual’s behavioral intention has a significant impact on his actual behavior which has been proved by abundant scholars’ research (Soorani and Ahmadvand, 2019; Zhang et al., 2019). In the past, some scholars used the measurement results of behavior intention to replace the measurement of actual behavior. However, this fuzzy substitution is controversial (Zhao et al., 2018, 2019). Therefore, one of the inspirations of TPB to this paper is that energy efficiency label should measure consumers’ purchase intention and purchase behavior separately.

At the same time, TPB emphasizes that individual’s behavior attitude significantly affects the occurrence of behavioral intention (Zhang et al., 2017), but the concept of behavioral attitude is too vague and difficult to measure effectively, so variables that are not easily confused and more easily measured can be selected for substitution (Paul et al., 2016; Scalco et al., 2018). For these reasons, this paper selects two variables: consumers’ cognition degree and trust degree on energy efficiency label to measure consumers’ behavior attitude.

In addition, TPB also shows that normative beliefs in the external social environment play a significant role in individual behavior intention (Ajzen, 1991). Although individual internal psychological factors are the important research objects of consumers’ purchase behavior of energy-saving appliances, we cannot ignore the influence of social environment variables on purchase intention and behavior. Therefore, this paper selects the subjective norm dimension in TPB framework to represent the external social environment variables in the purchasing situation.

To sum up, this paper makes the following improvements based on the basic framework of TBP theory. First, we measure consumers’ purchase intention and purchase behavior separately, and consider that purchase intention significantly affects purchase behavior. Secondly, consumers’ attitude toward energy efficiency label has impact on their intention when they buy energy-saving appliances. This paper uses two variables of consumer’s cognition and trust to define consumer’s behavior and attitude. Third, the external social environment variables (subjective norms) will have a significant impact on consumers’ purchase intention of energy-saving appliances.

#### Social Cognitive Theory

Previous studies of cognitive psychologists often ignore the effect of social environment variables on behavior. Albert Bandura, an American psychologist, proposed Social Cognitive Theory (SCT), which takes social factors into account (Bandura, 1977). The main contribution of SCT is the framework of ternary interaction theory, which includes three factors: individual, environment and behavior. It emphasizes the role of environment-individual-behavior interaction. Environmental factors include the whole social environment, such as political, economic, cultural and other environmental factors. Personal factors include individual cognition, motivation, attitude, and ability. The theory holds that, in the actual process of individual behavior, the environment and individual have the greatest influence on the behavior, and both act on the behavior together. And the environment and individual also affect each other, that is, the environment will affect the individual, and different individuals have more or less influence on the environment (Bandura, 1986, 1999, 2002, 2006).

According to the perspective of SCT, the joint influence of external environmental factors and individual internal factors should be considered in the study of consumers’ purchasing behavior of energy-saving appliances (Trotta, 2018). In particular, consumers have an accumulation about environmental protection and energy knowledge. The interaction of external social environment and individual cognition determine whether the individual’s actual purchase behavior occurs. When the individual’s environmental awareness is high, the external environmental factors will guide consumers to buy energy-saving household appliances with higher energy efficiency level. When the individual’s sense of responsibility and environmental awareness are low, external environmental factors will not have a significant impact on consumers’ actual purchase behavior (Bandura, 2002, 2006). This view is consistent with TBP theory (Valliere, 2017; Samah, 2018). TBP also emphasizes that individual behavior attitude (internal cause), behavior intention (internal cause), and subjective norm (external cause) work together on individual actual behavior. Therefore, referring to SCT, this paper selects the subjective normative dimension in TPB framework as the external social environment variables in the purchasing situation when studying the impact mechanism of energy efficiency label on consumers’ purchasing behavior of energy-saving appliances.

At the same time, publicity and education of environmental protection is also selected as one of the social environmental variables in this paper, because it is of great significance to solve environmental problems permanently (Ata, 2018; Zhang et al., 2021). Strengthening publicity and education of environmental protection can effectively improve residents’ awareness of environmental protection and energy conservation, and reduce household energy consumption (Yang et al., 2016; Emiru and Waktola, 2018; Yang, 2018). Many scholars have analyzed the impact of publicity and education on environmental behavior from the perspective of government intervention strategy. Some scholars clearly pointed out that the publicity of green consumption can not only convey the correct connotation of green consumption, but also improve consumers’ willingness to green consumption and the knowledge level of environmental protection (Bolderdijk et al., 2013). Based on cognitive learning theory, it is found that individual cognition of green consumption and environmental knowledge have a significant impact on promoting consumers’ purchase behavior of green products (Mohamed, 2007).

Finally, according to the viewpoint that environmental factors and individual factors jointly act on behavior in social cognitive theory, this paper constructs the theoretical model framework from the individual internal factors and social external factors that affect the purchase behavior of energy-saving appliances by energy efficiency label.

#### Signaling Theory

Under the situation of information asymmetry, Spence’s signal transmission theory holds that signal can transmit unobservable attributes to different individuals, so as to alleviate the phenomenon of information asymmetry (Spence, 2002). Signal transmission theory includes three core elements: signal, signal sender, and signal receiver. It solves the problems of the uncertainty of consumption market and labor market and information asymmetry between them as well as how to transfer unobservable attributes, such as trustworthiness, among individuals.

Since trustworthiness (e.g., product quality and certification) cannot be directly observed, we have to identify it by relevant external signals (Connelly et al., 2011; Cheung et al., 2014). Consumers who come into contact with a product for the first time often try to look for various signals related to product quality to infer the real quality of the product and its comparison with other products (Xu et al., 2013). Similar to the various information tips or signals attached to products, environmental labels are a tool used by consumers to evaluate the quality and environmental impact of products (Atkinson and Rosenthal, 2014; Issock et al., 2018). Atkinson and Rosenthal (2014) used ST to study the energy-saving behavior of 213 college students in the United States. In their study, environmental label is an important marketing signal and positively affects consumers’ trust in the green declaration of products. Issock et al. (2018) investigated the main drivers of consumers’ concerns about energy efficiency labels in South Africa using ST and behavior attitude theory, and the results show that when buying energy efficient appliances, consumers would pay attention to energy efficiency labels if they have confidence in energy-saving certified products. Although environmental labels are important product information, many consumers tend to ignore these signals when purchasing products (Tan et al., 2016), which may be due to lack of prominent environmental label information and understanding of environmental label (Thøgersen, 2000), or lack of trust in signals of green products (Liobikien et al., 2017).

This study supports ST. Energy efficiency labels can provide consumers with important information such as product attributes, energy efficiency grade and reliability, thus effectively reducing the asymmetry of product information and enhancing consumers’ product cognition or trust. Therefore, when choosing energy-saving home appliances, consumers tend to attach importance to energy efficiency label signals, perceive information such as the quality and price of energy-saving home appliances, which will influence their purchase intention and purchasing behavior.

### Variables Selection

The theoretical model of this paper is not completely expanded by adding variables under the framework of TPB theory. Its core idea is to build a theoretical model framework system based on the social cognitive theory, starting with the individual internal factors and social external factors that affect the purchase behavior of energy-saving appliances by energy efficiency labels. Then based on TPB, social cognitive theory and signal transmission theory, the theoretical model variables are selected through exploratory factor analysis. Through the exploratory factor analysis of 179 valid trial survey data, it is found that the perceptual behavior control variables in the TPB framework are not in good agreement with the data we collected, so they are considered and abandoned in our theoretical framework. The selection of variables is as follows. Firstly, according to TPB and signal transmission theory, the individual internal factors based on energy efficiency labels choose three factors: cognition, trust and perceived value based on energy efficiency labels. Secondly, according to TPB and social cognitive theory, publicity and education and subjective norms are selected to measure individual external environmental factors. In addition, it also focuses on the impact of consumers’ purchase intention on purchase behavior.

#### Individual Internal Factors

(1) Cognitive level. Cognition can be understood as the process of recognizing, selecting, organizing and explaining the stimuli acting on individuals. Generally, consumers only pay attention to stimuli closely related to their existing needs, beliefs and attitudes. So cognitive level is defined as consumers’ acceptance and interpretation of information stimuli related to external energy efficiency labels. Consumers selectively accept and interpret the stimuli related to energy efficiency labels according to individual characteristics. Therefore, there are differences in consumers’ cognition of energy efficiency labels. The degree of cognition mainly includes the following three aspects: consumers’ understanding of energy efficiency labels, consumers’ attention to energy efficiency labels and their perception of energy labels.
(2) Trust degree. Trust degree refers to consumers’ expectations on the energy efficiency and environmental information of products displayed on the energy efficiency labels (Chen et al., 2015). It mainly includes three aspects: consumers’ trust degree in the cognitive institutions of energy efficiency labels, consumers’ trust in the certification process of energy efficiency labels and consumers’ trust in the information marked on the energy efficiency labels.
(3) Perceived value. Perceived value mainly includes perceived quality and perceived price of energy-saving household appliances. Perceived quality refers to consumers’ overall evaluation of the excellence or superiority of products, which is affected by consumers’ impression in advance. Perceived price is considered to be the key determinant of purchasing environmental protection products. Consumers with strong awareness of energy conservation and environmental protection are usually willing to pay more for energy conservation and environmental protection products (Testa et al., 2015). Perceived value mainly includes three aspects: consumers’ perception of the quality of energy-saving household appliances, consumers’ perception of the price of energy-saving household appliances and consumers’ comprehensive perception of the value of energy-saving household appliances.

#### External Environmental Factors

(1) Publicity and education. Based on the research results of relevant scholars, publicity and education are used to represent one of the external environmental factors of society. Whether the government or household appliance enterprises publicize energy efficiency labels or energy-saving household appliances, it will have a certain impact on consumers’ energy-saving behavior. Publicity and education mainly consider the following three aspects. First, consumers take the initiative to publicize the willingness of energy-saving appliances. Second, consumers’ willingness to actively participate in publicity activities. Third, consumers’ acceptance level of publicity and education on energy-saving household appliances.
(2) Subjective norms. The framework of TPB considers the factors of subjective norms, takes into account the influence of others on consumers’ purchase behavior, and believes that subjective norms affect individuals’ actual behavior by affecting their behavior intention. Subjective norms mainly consider the following three aspects: first, people who are important to consumers want them to buy. Second, the cognition of important people to consumers’ purchase behavior. Third, their purchase behavior of people who are important to consumers.
(3) Purchase intention
  Purchase intention refers to the possibility that consumers give priority to environmental protection products rather than traditional products when considering purchase. Consumers’ attention to energy efficiency labels reflects consumers’ willingness and behavior in purchasing behavior to a certain extent. It mainly consider the following three aspects: first, consumers’ willingness to buy energy-saving appliances, second, consumers’ willingness to recommend others to buy energy-saving appliances, and third, consumers’ willingness to pay more for energy-saving appliances with higher energy efficiency.
(4) Purchase behavior
  Purchase behavior is defined as the actual purchase behavior finally taken by consumers. It mainly considers the following three aspects: the premium of energy-saving household appliances compared with ordinary household appliances, the actual consumption of energy-saving household appliances and the purchase frequency of energy-saving household appliances.

### Research Hypothesis

#### The Relationship Individual Internal Factors and Purchase Intention as Well as Purchasing Behavior

(1) The relationship between Individual internal factors of Energy Efficiency Labels and purchase intention as well as purchasing behavior
  Most of the research show that energy efficiency labels will generate purchasing intention for energy-saving appliances then lead to actual purchasing behavior (Wang Z. et al., 2019). Other studies on environmental labels such as carbon labels and ecological labels also show that environmental labels have a positive impact on consumers’ purchase intention and purchasing behavior (Issock et al., 2018). According to the previous theoretical basis and variable selection analysis of this study, the individual internal factors affecting the purchase behavior of energy efficiency labels mainly include three dimensions: consumers’ label cognition, label trust and perceived value based on energy efficiency labels.

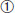
 **192Label cognition (LC)**

Energy efficiency labels provide consumers with more information about the energy efficiency of products. If consumers have higher awareness and more concerns about the energy efficiency labels, they will be more motivated to buy energy-saving household appliances (Shen and Saijo, 2009). A study from the United States shows that as of 2008, more than 75% of the total population have a certain awareness of “Energy Star,” and there is a rising trend. This increase in awareness will lead consumers to switch from non-Energy Star appliances to energy-saving appliances with the logo (Murray and Mills, 2011). It is obvious that consumers’ label cognition of home appliance products has a significant influence on consumers’ Purchase Intention (PI) and Purchasing Behavior (PB). Therefore, the following hypotheses are proposed:

H1a: Consumers’ perception of energy efficiency labels has a significant positive impact on their purchase intention of energy-saving appliances.

H1b: Consumers’ perception of energy efficiency labels has a significant positive impact on their purchasing behavior of energy-saving appliances.

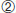
 **193Label Trust (LC)**

Consumers usually distrust environmental labels as they often suspect that “energy-saving features” makes deceptive assertions (Atkinson and Rosenthal, 2014). Studies have proved that the distrust of energy efficiency labels play a negative regulatory role in the purchase intention, which also shows that trust in energy efficiency labels can affect consumers’ purchase intention (Daugbjerg et al., 2014; Nuttavuthisit and Thøgersen, 2017). Some studies have proved that third-party certification is the most important driving force for buying green and energy-saving products. That is, consumers’ purchasing behavior is significantly affected by label trust (Ishak and Zabil, 2012; Topolansky Barbe et al., 2013). Therefore, the following hypotheses are proposed:

H2a: Consumers’ trust in energy efficiency labels has a significant positive impact on their purchase intention of energy-saving appliances.

H2b: Consumers’ trust in energy efficiency labels has a significant positive impact on their purchasing behavior of energy-saving appliances.

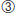
 **194Perceived value (PV)**

In this paper, perceived product quality and perceived product price are integrated into perceived value for measurement. According to the Signaling Theory, product price and product quality are important marketing signals, which can improve the perceived ability products. Besides, it also has impact on consumers’ purchase intention (Cheung et al., 2014). Previous studies have shown that consumers’ evaluation of product value largely depends on their perception of quality and price. Then the perceived value affects their purchase intention and purchasing behavior (Ariffin et al., 2016; Haryanto and Budiman, 2016; Liobikien et al., 2017; Marakanon and Panjakajornsak, 2017; Song et al., 2019b). Therefore, the following hypotheses are proposed:

H3a: Consumers’ perceived value based on energy efficiency labels has a significant positive impact on their purchase intention of energy-saving appliances.

H3b: Consumers’ perceived value based on energy efficiency labels has a significant positive impact on their purchasing behavior of energy-saving appliances.

(2) Relationship between individual internal factors

In addition to exploring the individual internal factors that affect the purchasing behavior, the relationship between individual internal factors should also be analyzed. When consumers understand the meaning of environmental labels, they will choose to trust the information annotated on the label (Issock et al., 2018). Therefore, label cognition plays a significant positive role in label trust. At the same time, consumers’ perceived value based on energy efficiency labels also significantly affects consumers’ trust in relevant information labels. A research survey on energy-saving electronic products in Thailand shows that green satisfaction and perceived quality positively affect green trust, and green perceived quality also partially mediates the positive relationship between environmental friendliness and green trust (Chen et al., 2016). Based on the above analysis, we propose the following hypotheses:

H4: consumers’ perception of energy efficiency labels has a positive and significant impact on their label trust in energy efficiency labels.

H5: consumers’ perceived value based on energy efficiency labels has a significant positive impact on their label trust in energy efficiency labels.

#### The Relationship Between External Environmental Factors and Purchase Intention

According to SCT, the factors influencing consumers’ behavior can be roughly divided into external environmental factors and internal psychological ones. The factors relevant to energy efficiency labels mainly achieve the purpose of influencing consumers’ purchasing behavior through the internal psychological factors of consumers. As one of the social environmental factors, external environment publicity and education can effectively improve human’s cognitive of environmental issues and promote the formation of residents’ awareness of energy conservation and environmental protection, thus influencing their purchasing behavior (Steg, 2008).

Many scholars have analyzed the impact of publicity and education on environmental behavior from the perspective of government intervention strategy. Some scholars clearly pointed out that the publicity of green consumption cannot only convey the correct connotation of green consumption, but also improve consumers’ willingness to green consumption and the knowledge level of environmental protection (Bolderdijk et al., 2013). Based on cognitive learning theory, it is found that individual cognition of green consumption and environmental knowledge play a significant role in promoting consumers’ purchase behavior of green products (Mohamed, 2007).

Meanwhile, TPB emphasizes that external social environmental factors such as subjective norm have a significant positive impact on consumers’ purchase intention (Wang Z. et al., 2019). Empirical results from India show that friends are a crucial factor which impact on consumers’ purchase decisions, that is, subjective normative factors in the TPB framework significantly affect consumers’ intention to buy energy-saving products (Testa et al., 2015). Therefore, in addition to considering the impact of energy efficiency labels on consumers’ purchasing behavior of energy-saving appliances, this paper also studies the influence of two external environmental factors, namely, Publicity and Education (PE) and Subjective Norm (SN). Here, we propose the following hypotheses:

H6: Publicity and education of energy-saving appliances have a significant positive impact on consumers’ purchase intention.

H7: Subjective norms have a significant positive impact on consumers’ purchase intention of energy-saving appliances.

#### The Intermediary Role of Purchase Intention

TPB emphasizes the positive influence of individual’s behavioral intention on his actual behavior. Studies have shown that the increase in consumers’ willingness to buy green products will positively promote the formation of green purchasing behavior (Trivedi et al., 2018). This means that green purchase intention is the most critical factor in green purchase behavior (Chen and Tung, 2014; Liobikien et al., 2017; Yadav and Pathak, 2017). At the same time, ecological labels, environmental value, and consumers’ knowledge of green products all significantly affect consumers’ purchase intention, thus further influencing consumers’ green purchasing behavior (Yadav and Pathak, 2017; Zhang et al., 2019). Combined with the framework of TPB, it can be found that purchase intention acts as an intermediary variable between behavior attitudes such as label cognition as well as label trust and purchasing behavior (Zhao and Zhong, 2015; Zhao et al., 2016).

In addition, perceived value not only affects consumers’ purchase intention, but also directly affects their purchasing behavior to a certain extent, while purchase intention affects their purchasing behavior. Therefore, purchase intention plays an intermediary role between perceived value and purchasing behavior (Sweeney and Soutar, 2001; Petrick, 2002; Clement et al., 2013). The following hypotheses are proposed in this paper:

H8: Consumers’ purchase intention of energy-saving appliances has a significant positive influence on their purchasing behavior.

H9a: Purchase intention plays an intermediary role in the influence of label cognition on purchasing behavior.

H9b: Purchase intention plays an intermediary role in the influence of label trust on purchasing behavior.

H9c: Purchase intention plays an intermediary role in the influence of perceived value on purchasing behavior.

**FIGURE 1:**
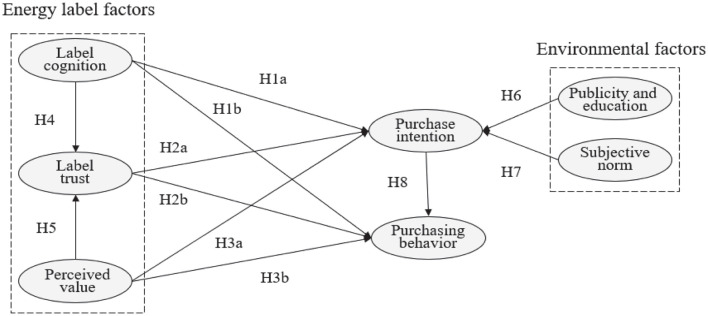
Proposed conceptual model.

Figure 1 is the theoretical framework model studied in this paper, which describes the hypothetical relationship between variables.

## Methodology

### Selection of the Research Method

At present, the situational experiment and questionnaire survey are the two main methods to investigate consumers’ purchasing behavior. Situational experiment is a method to create, control or change certain conditions or situations with a purpose to cause the psychological activities and behavior of the experimenters. Although this method can test the causal relationship between variables by actively creating or controlling experimental conditions, there are obvious defects (Lewis and Sjoestrom, 2010). Firstly, the simulated test scenario is not real, and it is difficult to design such a virtual artificial environment, and its novelty and external validity are not high; Secondly, the setting cost of scenarios is high and the period is long. Generally, small samples are taken as research objects without random sampling, and thus the research conclusions are not universal; Thirdly, the method mainly simulates the current and future situation, and is not applicable to the past experiences.

Therefore, in the light of the applicable conditions and limitations of the situational experiment, this paper adopts the method of questionnaire survey. As a common method in market survey, the core of this method is questionnaire design and questionnaire survey. This method can achieve large random sampling in a short period of time, obtain a large number of extensive data and information and ensure the scientific nature of the research results.

### Questionnaire Structure

The questionnaire is composed of two parts. The first part is demographic characteristics information, including five identification questions such as gender, age, education level, job category, and family annual income level. The second part consists of seven variable questions, involving 21 research questions, among which six variables, namely, perceived value, label cognition, label trust, subjective norm, publicity, and education as well as purchase intention are measured by Likert five-point scale. The purchasing behavior is mainly measured by three research questions, which aim to reflect the actual purchasing behavior of energy-saving appliances. The purchase frequency and purchase intention of energy-saving household appliances are also measured by Likert five-point scale, and the price proportion range of energy-saving appliances higher than ordinary appliances is measured by percentage range. All the questions in the questionnaire are modified by the mature scale or the results of existing studies. In order to ensure the validity of the scale, 30 participants are selected from the population to conduct a preliminary test. In the preliminary test, ambiguous and repetitive questions are deleted. Then the reliability of the scale is evaluated, and it showed acceptable levels of internal consistency according to their Cronbach’s alphas.

### Sample Selection

In order to study the influence of different factors on consumers’ purchasing behavior of energy-saving appliances, it is necessary for consumers to determine their perception of these internal and external factors according to their own purchase experience. In order to ensure the representativeness of the sample, considering that the student group and the group under 20 years old do not have the demand for energy-saving appliances and the probability of active purchase is not high, the two groups are excluded in the sample selection. The paper questionnaires are distributed to major shopping malls in the main urban area of Mianyang City, China. In this paper, data are collected by intercepting interview method and consumers who voluntarily participate in the questionnaire survey are selected by professional trained staff in the shopping area of household appliances. The questionnaire survey personnel observed the consumers who entered the home appliance purchase area, and selected the consumers who have consulted the merchant’s product information or have purchased home appliances as the survey object. Ask consumers if they are willing to participate in the questionnaire without causing resentment. If so, guide them to the workbench where the survey team is located to fill in the questionnaire. If not, investigators also need to thank. The consumers who participated in the questionnaire survey are all voluntary and the research group do not provide any form of reward. A total of 425 questionnaires were distributed, and 396 valid questionnaires and 29 invalid questionnaires were recovered with the questionnaire validity of 92%. During the pre-processing of the survey data, there were 29 questionnaires with many missing data and completely consistent questionnaire data, and they were excluded from the recovered questionnaire.

## Results

### Test on Common Method Variance

The problem of Common Method Variance (CMV) is likely to arise by using the questionnaire data (Chang et al., 2010). The Harman single factor method is usually used to test CMV, that is, factor analysis is performed on all items in the questionnaire, and the first principal component proportion without rotation is solved, which reflects the quantity of CMV (Gorrell et al., 2011). When not rotated, the factor load of the first principal component is 39%, lower than 40% of the standard value, indicating that CMV results are within the acceptable range, and thus the test on CMV is passed.

### Descriptive Statistics of the Study Sample

Table 1 describes the descriptive statistics of respondents. From the perspective of gender dimension, the frequency of male sample (52%) is slightly higher than that of female sample (48%), and the distribution of gender characteristics is generally balanced. From the perspective of age group, the samples are mainly young and middle-aged. The cumulative frequency of samples aged between 21–50 years old reaches 94.44%. The education level of the sample is mainly distributed in senior high school or technical secondary school (27%), junior college (11%), and undergraduate (29%), which conforms to the current educational structure of China. From the perspective of household annual income, the three sample groups of below 80,000 yuan (28%), 80,000–150,000 yuan (35%), and 150,000–400,000 yuan (34%) shows a balanced distribution, with the cumulative proportion reaching 97%. This study collected 396 valid questionnaires. The sample size is far more than 10 times the number of parameters to be estimated by the model (Hoogland and Boomsma, 1998), and he sample size generally obeys the normal distribution, so it is reasonable. In the sample data, the absolute value of skewness of all indicators is less than 2, and the absolute value of skewness of most indicators is close to 0; the absolute value of kurtosis of all indicators is less than 3. This means that the sample data follows the normal distribution, the distribution of different characteristics of the sample can represent the different characteristics of current consumption of energy-saving household appliances, so the sample data is representative.

**TABLE 1:** Demographic profile of respondents.

Variables		N	Percentage
Gender	Male	206	52%
	Female	190	48%
Age group	21–30	72	18%
	31–40	149	38%
	41–50	153	39%
	Above50	22	6%
Education level	Primary school	73	18%
	High school	108	27%
	Junior college	44	11%
	Undergraduate	114	29%
	Postgraduate	57	14%
Household annual	0–80 K	111	28%
Income (RMB)	80–150 K	140	35%
	150–400 K	134	34%
	400–800 K	8	2%
	Above 800 K	3	1%

Before testing each hypothetical model, the correlation analysis of each variable should be carried out firstly. The mean, standard deviation and correlation coefficients of the seven variables involved in this study are shown in Table 2. The results showed that all correlation coefficients were significantly positively correlated.

**TABLE 2:** Variable mean, standard deviation, and correlation coefficient.

	Label cognition	Label trust	Perceived value	Publicity and education	Subjective norm	Purchase intention	Purchasing behavior
Mean	3.91	3.78	3.56	3.74	3.60	4.05	2.83
S.D	0.86	0.82	0.84	0.79	0.83	0.82	1.05
Label cognition	1						
Label trust	0.52**	1					
Perceived value	0.36**	0.47**	1				
Publicity and education	0.49**	0.48**	0.35**	1			
Subjective norm	0.36**	0.46**	0.43**	0.50**	1		
Purchase intention	0.43**	0.53**	0.52**	0.51**	0.54**	1	
Purchasing behavior	0.13**	0.29**	0.35**	0.19**	0.28**	0.38**	1

“**” means P < 0.01.

### Model Testing

KMO test and Bartlett’s spherical test are required for the variables in the scale with the help of SPSS software analysis tools. The results show that the KMO test value is 0.85, which is greater than the critical value of 0.50; the concomitant probability of Bartlett’s spherical test is 0.00, less than 0.05. This means that the items of the scale are suitable for factor analysis. Therefore, through the exploratory factor analysis (EFA) results, the total contribution rate of the sum of rotation squares of the seven factors reaches 77%, and the factor loading values after orthogonal rotation are between 0.5 and 0.95, which meets the parameter test conditions.

Then, the reliability and validity of the scale need to be tested, and the results are shown in Table 3. Reliability analysis is required to verify the consistency or reliability of the test results so as to verify the authenticity of the sample data. The composite reliability (CR) and Cronbanch’s α coefficient are both greater than 0.70, indicating that the scale has good reliability (Wang B. et al., 2019). The validity is the explicit variable to measure the validity and accuracy of latent variables, and the convergent validity is usually used to judge whether the model is valid or not. It can be seen from Table 3 that the average variance extracted (AVE) of each variable is greater than 0.50, which means that the structural equation model in this paper has passed the convergence validity test (Fornell and Larcker, 1981).

**TABLE 3:** Reliability test results of the scale.

Dimension	Average variance extracted (AVE)	Composite reliability (CR)	Cronbanch’s alpha
Label cognition	0.65	0.85	0.85
Label trust	0.78	0.91	0.92
Perceived value	0.56	0.79	0.79
Publicity and education	0.51	0.76	0.77
Subjective norm	0.68	0.86	0.86
Purchase intention	0.74	0.88	0.90
Purchasing behavior	0.67	0.86	0.84

Finally, the model fitting should be tested overall, and the results are shown in Table 4. The absolute fitting indicators meet the fitting criteria, among which, χ2/df (1.99) is less than the critical value 2, AGFI is greater than 0.9, and RMSEA (0.05) is less than 0.08. Two value—added fitting indicators, CFI and TLI, are also up to the standard. This means that the measurement model and its parameter estimation are valid and the fitting effect of the measurement model is good.

**TABLE 4:** Model fitting result table.

Fitting indicator	χ ^2^/df	GFI	AGFI	CFI	TLI	RMSEA
Test result	1.99	0.93	0.90	0.97	0.96	0.05
Judging criteria	<2	>0.90	>0.90	>0.90	>0.90	<0.08
Model fitting judgment	Yes	Yes	Yes	Yes	Yes	Yes

### Hypothesis Test Results

**FIGURE 2:**
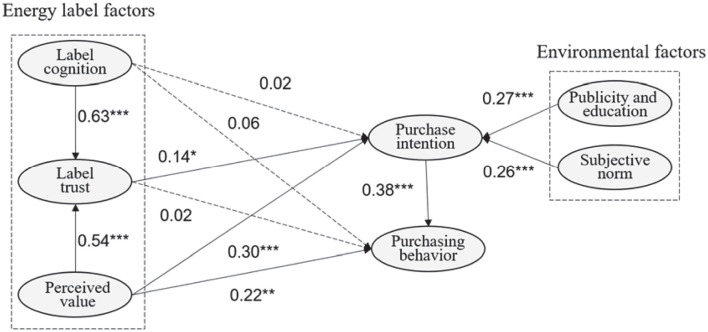
Structural model. “*” means *P* < 0.05, “**” means *P* < 0.01, “***” means *P* < 0.001, “ns” means *P* > 0.05.

The structural equation model is used to test the influence mechanism of factors related to energy efficiency labels on consumers’ purchasing behavior of energy-saving appliances. The test results are shown in Table 5 and Figure 2. In addition to label cognition, label trust, perceived value, publicity and education as well as subjective norm all have positive effects on consumers’ purchase intention of energy-saving appliances, that is, hypothesis H1a is untenable, hypothesis H2a, H3a, H6, H7 are all tenable.

**TABLE 5:** Hypothesis test results.

Hypothesis	Regression path	Standardized path coefficients	*P*-value	Results
H1a	LC→PI	0.02	ns	Rejected
H1b	LC→PB	−0.06	ns	Rejected
H2a	LT→PI	0.14	*	Supported
H2b	LT→PB	0.02	ns	Rejected
H3a	PV→PI	0.30	***	Supported
H3b	PV→PB	0.22	**	Supported
H4	LC→LT	0.63	***	Supported
H5	PV→LT	0.54	***	Supported
H6	PE→PI	0.27	**	Supported
H7	SN→PI	0.26	***	Supported
H8	PI→PB	0.38	***	Supported
H9a	LC→PI→PB	−	−	Rejected
H9b	LT→PI→PB	−	−	Supported
H9c	PV→PI→PB	−	−	Supported

“*” means P < 0.05, “**” means P < 0.01, “***” means P < 0.001, “ns” means P > 0.05.

Label cognition and label trust do not directly affect consumers’ purchasing behavior, while perceived value and purchase intention significantly affect purchasing behavior, and the influence of purchase intention on purchasing behavior is significantly greater than that of perceived value. In other words, hypothesis H1b and H2b are not tenable, while hypothesis H3b and H8 are. Both label cognition and perceived value have positive effects on consumers’ trust for energy efficiency labels, proving that H4 and H5 are tenable. From the intermediary effect results of structural equation, the hypothesis that purchase intention, as the intermediary variable of label trust and perceived value affects consumers’ purchasing behavior has passed the test, that is, H9b and H9c have been verified, while purchase intention as the intermediary variable of label cognition influencing purchasing behavior has not been verified, meaning that H9a does not hold.

To further verify the significance of the intermediary effect, this paper uses Process3.2 to test the intermediary effect of purchase intention between label cognition, label trust as well as perceived value and purchasing behavior. The test results are shown in Table 6. The indirect effect in the table represents the intermediary effect, and if the indirect effect is significant, there is the intermediary effect. From the parameter estimates, the intermediary effect of label trust and perceived value are 0.16 and 0.29, respectively. The deviation correction Bootstrap confidence intervals under 95% confidence level are [0.02, 0.30] and [0.14, 0.43], respectively, excluding the 0 value. This indicates that purchase intention has a significant intermediary effect between label trust, perceived value and purchasing behavior, that is, H9b and H9c are valid. The estimated intermediary effect of label cognition is 0.05, and the deviation correction Bootstrap confidence interval under 95% confidence level is [−0.17, 0.08], including the 0 value, that is, the intermediary effect is not significant, and H9a is not tenable. In a word, the consistency between the results of intermediary effect and that of structural equation is further tested by process.

**TABLE 6:** Intermediary effect test results.

Regression path	Indirect effect	Standard error	t	*p*	Bootstrap’s 95% CI	
					LLCI	ULCI
LC→PI→PB	0.05	0.06	0.72	0.47	−0.17	0.08
LT→PI→PB	0.16	0.07	2.26	0.03	0.02	0.30
PV→PI→PB	0.29	0.07	3.96	0.00	0.14	0.43

According to the hypothesis test results of the structural equation model, the influence mechanism model of energy efficiency labels on consumers’ purchasing behavior of energy-saving appliances is shown in Figure 2. It is easy to find that among the relevant factors of energy efficiency labels; the cognition degree and perceived value of energy efficiency labels jointly affect the trust degree. The trust degree and perceived value affect consumers’ actual purchasing behavior through the intermediary effect of purchase intention, while the perceived value can directly influence purchasing behavior. Therefore, the related factors of energy efficiency labels are not simple coordinate relations. External environmental factors such as publicity and education as well as subjective norms also influence consumers’ actual purchasing behavior through the intermediary role of purchase intention.

## Discussion

This study discusses the impact of energy efficiency labels on consumers’ purchase intention and purchase behavior of energy-saving household appliances from two aspects: individual internal factors and external environmental factors. The individual internal factors include behavior attitude of TPB framework and perceived value of signal theory. And behavioral attitude variables are subdivided into two dimensions: label cognition and label trust. The external environmental factors include publicity and education and subjective norms.

Previous studies have confirmed that carbon label cognition has a significant positive impact on label trust (Zhao et al., 2017). Our results further verify this result, that is, the standardized regression coefficient of the path from label cognition to label trust is 0.63, which is significant at the level of 0.001. Label recognition is one of the preconditions for the establishment of label trust. The higher consumers’ awareness of energy efficiency labels, the more they trust energy-saving household appliances with higher energy efficiency levels (Wang B. et al., 2019). The standardized estimated coefficient of the path from perceived value to label trust is 0.54 and passes the test of significance level of 0.001. That is, the higher the perceived value level of energy efficiency labels, the higher the label trust level. Some studies pointed out that green perceived value has a positive impact on green trust when studying the purchase behavior of energy-saving electronic products (Chen et al., 2016), which is the same as our research results. This means that the perceived product price and the perceived product quality are the key influence signals to promote consumers’ trust in green products (Issock et al., 2018).

The empirical results show that label cognition has no significant effect on purchase intention and purchase behavior, but indirectly affects purchase intention and further affects purchase behavior through the intermediary variable of label trust (Wang B. et al., 2019). The standardized regression coefficient of the path from label trust to purchase intention is 0.14, which is significant at the level of 0.05. This means that the higher the trust level of energy efficiency labels, the stronger the consumers’ willingness to buy energy-saving household appliances with higher energy efficiency levels. This conclusion is consistent with the research results of most scholars (Wang B. et al., 2019), which further verifies the correctness of the ternary interaction model of social cognitive theory. There is no significant relationship between label trust and purchase behavior, but the intermediary effect test shows that the trust degree causes consumers’ attention to energy efficiency labels through the intermediary variable of purchase intention, resulting in purchase behavior (Paul et al., 2016). The standardized regression coefficients of the path from perceived value to purchase intention and perceived value to purchase behavior were 0.30 and 0.22, respectively, and both passed the significance level test of 0.001. This means that perceived value has a significant positive impact on purchase intention and purchase behavior. It shows that the higher the perceived value of energy-saving household appliances based on energy efficiency labels, the stronger the consumers’ willingness and behavior to buy energy-saving household appliances (Issock et al., 2018). Consumers perceive that the greater the environmental value of energy-saving appliances through energy efficiency labels, the more they feel that their value matches the price. At this time, the more obvious the positive bias of willingness and behavior to buy energy efficiency labeled products will be (Ariffin et al., 2016; Liobikien et al., 2017).

The standardized regression coefficients of the path from publicity and education to purchase intention and subjective norm to purchase intention were 0.27 and 0.26, respectively, and both passed the significance level of 0.001. Subjective norms indirectly affect purchase behavior through purchase intention (Wang Z. et al., 2019), which is also consistent with the TPB theoretical framework system (Ajzen, 1991). The publicity and education on energy efficiency labels has a significant positive impact on purchase intention, which means that publicity and education can effectively promote the formation of consumers’ environmental awareness, so as to generate positive purchase intention (Steg, 2008).

The standard regression coefficient of the path from purchase intention to purchase behavior is 0.34, which is significant at the level of 0.001. The results show that the purchase intention significantly affects the actual purchase behavior, that is, the stronger the purchase intention of consumers for energy-saving appliances, the greater the possibility of purchase behavior. This conclusion is not only consistent with the core view of TBP, but also consistent with other research conclusions, that is, green purchase intention is the most key leading factor of green purchase behavior (Chen and Tung, 2014; Liobikien et al., 2017; Yadav and Pathak, 2017; Trivedi et al., 2018; Zhang et al., 2019).

## Conclusion, Policy Implications, Limitations, and Perspectives

### Conclusion

This study constructs a theoretical model of the impact mechanism of energy efficiency labels on consumers’ purchase behavior by using social cognition theory, planned behavior theory and signal transmission theory. And the empirical test conclusions are as follows.

Firstly, both the label cognition and perceived value significantly affect label trust, which shows that label trust plays an important role in promoting purchase intention and purchase behavior. Therefore, it is very important to improve the understanding, attention and cognition of energy efficiency labels, and to provide consumers with important information such as product attributes, energy efficiency grades and reliability through the real energy efficiency labels information of products. When consumers buy energy-saving appliances, they often pay attention to energy efficiency labels signals, perceive the information such as the quality and price of energy-saving appliances products, thus enhancing consumers’ trust in energy-saving and energy-saving products, and improving consumers’ purchasing willingness and purchase behavior.

Secondly, among the factors related to energy efficiency labels, only perceived value has a direct positive impact on consumers’ purchasing behavior of energy-saving household appliances. Neither label cognition nor label trust has a direct effect on consumers’ purchasing behavior of energy-saving appliances, but influences consumers’ purchasing behavior through the intermediary variable—purchase intention. Through the intermediary effect test of purchase intention, it is found that purchase intention, as a complete intermediary variable, transmits the influence of energy efficiency label trust on purchasing behavior, while purchase intention, as a partial intermediary variable, transmits the influence of consumers’ perceived value for energy efficiency labels on the purchasing behavior of energy-saving appliances. This conclusion indicates the importance of consumers’ purchase intention on their actual purchasing behavior, and further reveals the connection mechanism of the factors related to energy efficiency labels on consumers’ purchasing behavior.

Thirdly, external environmental factors, such as publicity, education and subjective norm, have a significant impact on consumers’ intention to purchase energy-saving appliances, and affect actual purchasing behavior through the intermediary role of purchase intention. This conclusion mainly considers the influence of external environmental factors. The response mechanism of energy efficiency labels to consumers’ purchasing behavior of energy-saving appliances is not an internal closed mechanism, but a dynamic mechanism affected by external environmental variables. The publicity and education of energy-saving appliances and subjective norms have a significant impact on consumers’ purchase intention, which provides a new idea for promoting the formation of consumers’ purchasing behavior of energy-saving appliances.

### Policy Implications

According to the above research conclusions, it puts forward the following three policy implications.

(1) Improve consumers’ cognitive ability of energy efficiency labels. Although the energy efficiency labeling system has been popularized in China for more than 10 years, some consumers still do not understand energy efficiency label at all. The results show that consumers’ cognitive ability of labels significantly affects their trust in energy efficiency labels, thus affecting their purchase intention. So it is very important to improve consumers’ cognitive ability of energy efficiency labels. Therefore, the government and household appliance enterprises should do a good job in the popularization of energy efficiency label and root the concept of energy efficiency label in the hearts of consumers. At the same time, enterprises should enhance the credibility of energy efficiency label from the perspectives of energy efficiency labeling information, energy efficiency labeling certification institutions and transparency of energy efficiency labeling certification process, so as to leave a good impression on consumers.
(2) Enhance the perceived value of energy-saving household appliances through innovation. Consumers’ perceived value of energy efficiency labels significantly affects their willingness to buy energy-saving household appliances, and it is the only variable that can have a direct effect on purchase behavior. Therefore, we must recognize the importance of improving enterprise brand. First, household appliance enterprises should speed up technological innovation and product innovation, improve the quality of energy-saving products or promote product upgrading, and optimize and adjust the product structure. At the same time, they should rely on technological innovation to improve the control ability of the production process, improve the product qualification rate and the efficiency of comprehensive utilization of resources, and gradually improve the technical guarantee system. Secondly, focusing on consumer demand and its changes, they comprehensively should adopt various forms such as management innovation, organizational innovation, and value innovation to improve the image value of household appliances, improve loyalty and purchase confidence of consumers, so as to improve customer perceived value.
(3) Enhance consumers’ subjective norms through the publicity and education of energy efficiency labels. The governments and enterprises can make full use of traditional and emerging media to effectively transmit energy efficiency labeling information to the public, so as to improve consumers’ awareness of energy efficiency label. And home appliance enterprises should take user experience as the guidance, actively improve product service quality, form a good reputation effect, improve the subjective norms of consumers.

### Limitations and Perspectives

This study constructs and tests the formation mechanism of energy efficiency labels on consumers’ purchase behavior through empirical analysis, which has certain academic value and practical significance. But there are also some limitations.

Firstly, there is still room for improvement in the selection of research objects. The research object of this study is limited to the urban area of Mianyang, and the differences of economic development, social environment and other factors in different regions are not fully considered. There may be the problem of insufficient sample representation, especially the lack of samples from rural areas. Therefore, more extensive sampling can be adopted in more areas for follow-up research to expand the scope of application of the research.

Secondly, due to the constraints of research time, cost and other factors, this study uses time cut-off data to study the impact mechanism. Compared with long-term tracking research, the persuasion is relatively weak, which is difficult to reflect the dynamic process of the impact mechanism of energy efficiency labels on consumers’ purchase behavior. Therefore, the follow-up research can improve the persuasiveness of the empirical test results of the model through long-term tracking observation.

Thirdly, although the formation mechanism of energy efficiency labels on consumers’ purchase behavior is revealed from individual internal factors and external environmental factors, the demographic characteristics and consumers’ personal psychological characteristics affecting consumers’ purchase behavior are not considered. Therefore, the follow-up study can explore the influence mechanism of different factors on consumers’ purchase behavior.

## Data Availability Statement

The original contributions presented in the study are included in the article/supplementary material, further inquiries can be directed to the corresponding author/s.

## Author Contributions

GS-D and LC-P: conceptualization, writing—review and editing. GS-D, LH, and ZN: methodology, investigation, and writing—original draft preparation. LH and ZN: formal analysis. All authors have read and agreed to the published version of the manuscript.

## Conflict of Interest

The authors declare that the research was conducted in the absence of any commercial or financial relationships that could be construed as a potential conflict of interest.

## Publisher’s Note

All claims expressed in this article are solely those of the authors and do not necessarily represent those of their affiliated organizations, or those of the publisher, the editors and the reviewers. Any product that may be evaluated in this article, or claim that may be made by its manufacturer, is not guaranteed or endorsed by the publisher.
